# Gas Gangrene of the Spleen Caused by Clostridium perfringens After Mild Blunt Trauma

**DOI:** 10.7759/cureus.57429

**Published:** 2024-04-01

**Authors:** Akira Takeuchi, Yusuke Taki, Kento Furuya, Kenta Ito, Makoto Suzuki, Shinsuke Sato, Masaya Watanabe, Ko Ohata, Hideyuki Kanemoto, Noriyuki Oba

**Affiliations:** 1 Department of Gastroenterological Surgery, Shizuoka General Hospital, Shizuoka, JPN; 2 Department of Clinical Laboratory Medicine, Shizuoka General Hospital, Shizuoka, JPN; 3 Department of Pathology, Shizuoka General Hospital, Shizuoka, JPN

**Keywords:** blunt trauma, splenectomy, gas gangrene, clostridium perfringens, splenic abscess

## Abstract

Splenic gas gangrene caused by *Clostridium perfringens *is rare. A 73-year-old woman was referred to our hospital because of fatigue, dyspnea, and left hypochondrial pain. She had a history of blunt trauma to the left abdomen eight days ago. She presented with hypoxemia and a high inflammatory response on blood tests. A CT showed left pleural effusion and gas in the spleen. She was treated with antimicrobials and underwent splenectomy. *C. perfringens* was identified from blood and intraoperative ascites cultures. She recovered and was discharged on day 34 of hospitalization. As *C. perfringens* is part of the normal gut microbiota and can translocate to other parts of the body, this bacterium should be considered a splenic abscess pathogen when an intracorporeal anaerobic environment is suspected.

## Introduction

*Clostridium perfringens* is an anaerobic, gram-positive, rod-shaped, and spore-forming bacterium. *C. perfringens* is widely distributed in soils, marine sediments, sewage, and the gastrointestinal tract of humans and animals [1]. It is a common cause of food poisoning and soft-tissue infections known as gas gangrene or clostridial myonecrosis. Gas gangrene is a life-threatening condition that requires early treatment, including antibiotic administration and debridement [2]. Conversely, splenic abscess is a rare disease caused by a variety of pathogens, including gram-positive cocci, gram-negative rods, anaerobes, and Candida species [3]. Although *C. perfringens* is a normal human intestinal flora, reports of splenic gas gangrene are scarce. To the best of our knowledge, there are only seven case reports of splenic abscess caused by *C. perfringens*. Here, we report a case of splenic gas gangrene caused by *C. perfringens* after mild blunt trauma.

## Case presentation

A 73-year-old Japanese woman was referred to our hospital because of five days of fatigue, two days of dyspnea, and left hypochondrial pain. Eight days before her visit, she had fallen indoors and sustained blunt-force trauma to the left abdomen. Her past medical history was significant for appendectomy, hyperlipidemia, hypertension, and arteriosclerosis obliterans. Examination in the emergency department revealed hypoxemia with oxygen saturation (SpO2) of 89% and an elevated inflammatory response with a white blood cell count of 31,700/μL and a C-reactive protein of 39.26 mg/dL. Her CT scan showed pleural effusion and atelectasis in the left chest. She was admitted to our hospital with a diagnosis of pneumonia and pleural effusion. She was given meropenem owing to her allergy to a lot of antibiotics, including amoxicillin, cefditoren pivoxil, cefcapene pivoxil, garenoxacin mesylate, and levofloxacin.

**Figure 1:**
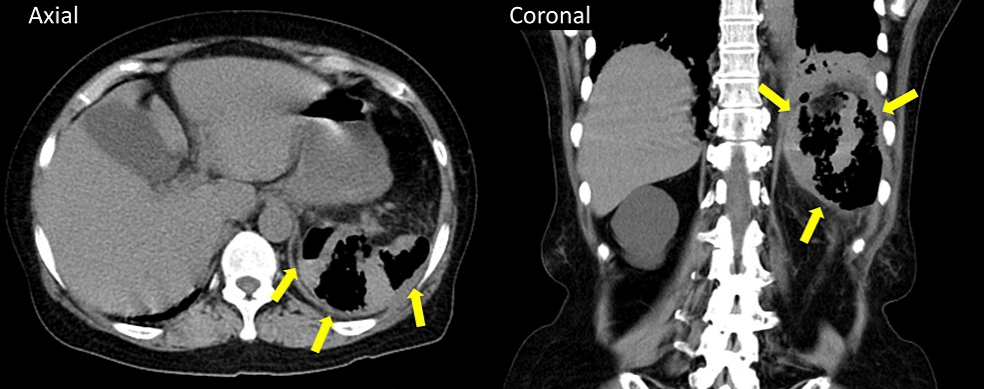
The axial and coronal planes of the CT show gas gangrene of the spleen Yellow arrows indicate gas in the spleen.

On day two of hospitalization, the radiologist noted gas gangrene of the spleen on the CT in the emergency department (Figure 1), and she was referred to gastroenterological surgeons. As the spleen was full of gas without fluid retention, which was suspicious of abscess, percutaneous puncture was not considered adequate. Therefore, an emergency splenectomy was performed on the same day. At laparotomy, the spleen was adherent to the diaphragm and surrounded by dark red blood. After ligation of the splenic artery and vein, the spleen was removed, and a drain was placed. The operative time was 191 minutes, and the intraoperative blood loss was 740 mL. Only *C. perfringens* was detected in the blood culture obtained in the emergency room, and the ascites were removed during surgery. Susceptibility testing revealed that *C. perfringens* was susceptible to many antibiotics, including penicillin, metronidazole, and meropenem. Because she was allergic to amoxicillin, the antibiotics were de-escalated to metronidazole and continued until day 13 of hospitalization. During the postoperative course, her respiratory function deteriorated immediately after surgery, requiring nasal high-flow oxygen administration, but it gradually improved and no longer required oxygen on day 12 of hospitalization. She started the oral diet on day seven and was discharged on day 34 of hospitalization after treatment and rehabilitation for arteriosclerosis obliterans. She was vaccinated against *Streptococcus pneumoniae* as an outpatient.

**Figure 2:**
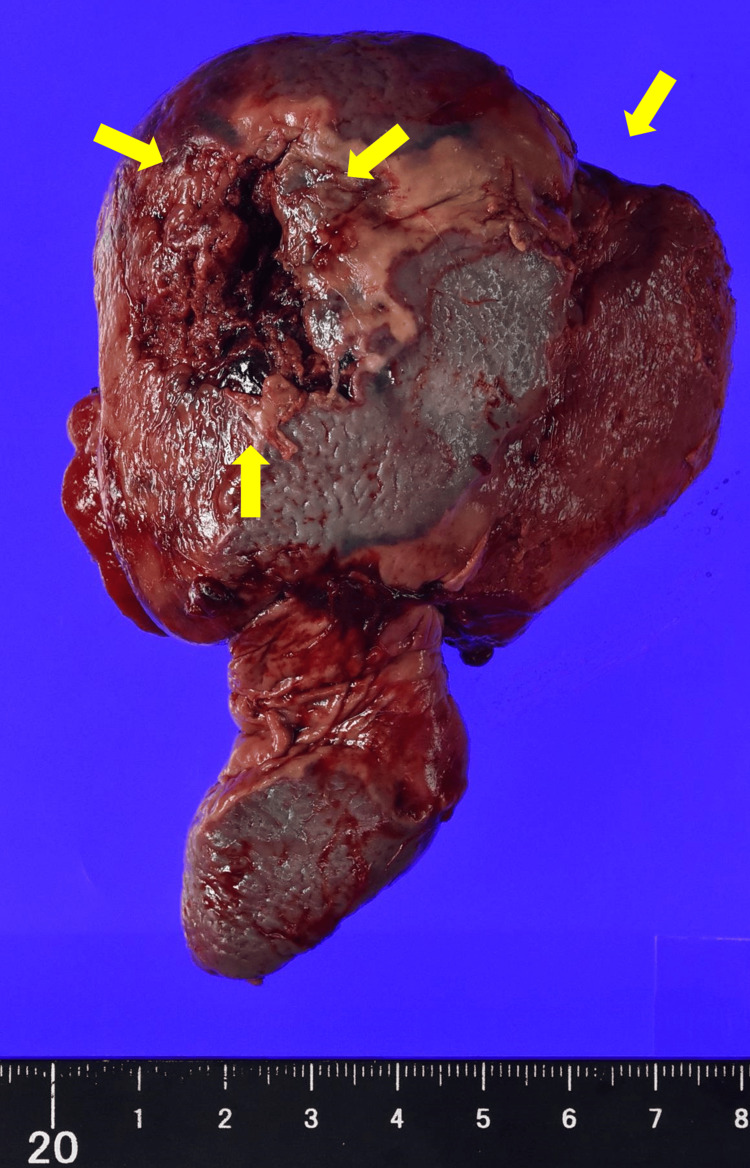
Gross picture of the surgically removed spleen There were many cavities that were suspected to have been filled with gas, and the serous membrane was perforated (yellow arrows).

**Figure 3:**
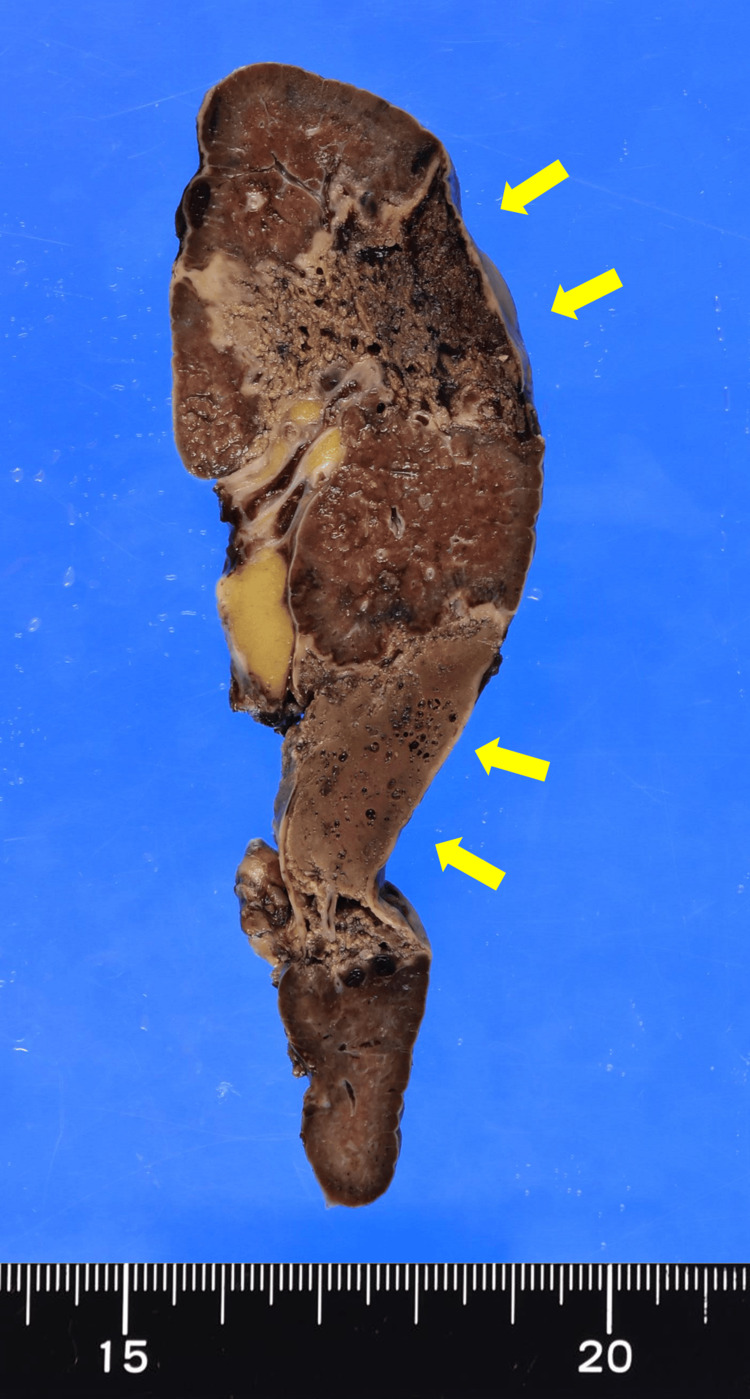
Cut surface of the spleen after formalin fixation Many cavities were observed in the infarcted area (yellow arrows).

**Figure 4:**
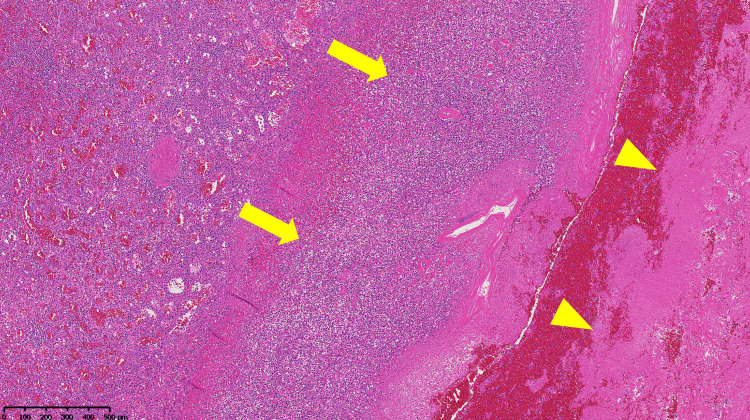
Microscopic picture of the surgically removed spleen Microscopic examination showed pyogenic inflammation (yellow arrows) and necrosis (yellow arrowheads) without abscess formation.

Pathological examination of the spleen showed irregular infarction and small cavities, which macroscopically represented gas production (Figures 2-3). Microscopic examination showed pyogenic inflammation and necrosis without abscess formation (Figure 4).

## Discussion

We report a case of splenic gas gangrene caused by *C. perfringens* after blunt trauma to the left abdomen. This case report is impressive in two respects. First, gas gangrene of the spleen itself is a rare cause of splenic abscess. Second, although this patient had a history of trauma, there is no skin laceration from which *C. perfringens* entered the body.

Splenic abscesses are rare, with an incidence of 0.14-0.7% reported in autopsy studies [4,5]. They usually occur in patients with splenic trauma, malignancy, diabetes mellitus, immunodeficiency disorders, or systemic infection [6]. Brook et al. reported 56 pathogens from 29 splenic abscesses; 23 were aerobic and facultative species, 31 were anaerobic or micro-aerophilic streptococci, and two were *Candida albicans* [3]. The predominant anaerobic pathogens were *Peptostreptococcus *spp., *Bacteroides *spp., and *Clostridium *spp. Although they reported two cases of *C. perfringens* in their bacterial profile, there was no detailed case report. We searched PubMed from 1946 to 2023 using the keywords "Clostridium perfringens" and "spleen" and found only seven cases of splenic abscess because of *C. perfringens* [7-13] (Table 1).

**Table 1:** Previous reports of splenic abscess by Clostridium perfringens *immunosuppression

Authors, publication year	Age	Sex	Comorbidity	Other bacteria	Treatment	Outcome
Gangahar et al., 1981 [7]	75	Male	Sickle cell trait	None	Splenectomy	Alive
Kitterer et al., 2014 [8]	71	Male	Liver transplant, IS*	None	Laparotomy	Dead
Dumas et al., 2017 [9]	48	Male	None	None	Splenectomy	Unknown
Meyer et al., 2019 [10]	50	Male	Patent foramen ovale, cerebral ischemia	*Streptococcus gallolyticus*, *Clostridium baratii*	Splenectomy	Alive
Oskutis et al., 2021 [11]	50	Female	Myelofibrosis, IS*	None	Drainage, splenectomy	Alive
Hellinckx et al., 2021 [12]	74	Female	Psoriatic arthritis, IS*	None	Drainage	Alive
Gafumbegete et al. 2021 [13]	29	Female	37 weeks' gestation	None	Laparotomy	Dead
Our case, 2024	73	Female	Arteriosclerosis obliterans, trauma	None	Splenectomy	Alive

Most cases of splenic gas gangrene had underlying disease and were infected with *C. perfringens* alone. Only the case reported by Gangahar et al. had a history of trauma [7]. Their case had been struck in the left side of the abdomen two weeks before admission. Our case is also struck in the left side of the abdomen; however, there is no skin laceration. The presumed pathophysiology of our case is as follows. The blunt trauma created a hematoma, an anaerobic environment within the spleen. Then, *C. perfringens* translocated from the intestine to the spleen and grew in the anaerobic condition. The increased *C. perfringens *produced alpha-toxin, which hydrolyzed cell membrane phospholipids, eventually leading to cell necrosis [14]. Alpha-toxin has been reported to play three roles in gas gangrene. First, it may interfere with the recruitment of immune cells, such as neutrophils, to infected tissues, potentially reducing pathogen clearance at the site of infection. Second, it can cause constriction of blood vessels, resulting in reduced blood supply to tissues, creating an anaerobic environment and promoting overgrowth of *C. perfringens*. Third, it can activate inflammatory cascades in host cell metabolism. Experiments in mice have shown that alpha-toxin increases serum inflammatory cytokines such as tumour necrosis factor alpha (TNF-α), IL-1β, and IL-6 [15]. The extreme leukocyte elevation in our case may result from such cytokine elevation.

Most cases of splenic abscess because of *C. perfringens* have required splenectomy. Two cases reported by Kitterer et al. and Gafumbegete et al. underwent laparotomy but could not receive any surgical treatment because of fatal conditions and passed away just after or during surgery [8,13]. Additionally, the case reported by Oskutis et al. improved temporarily with percutaneous drainage and antibiotics but recurred and required splenectomy [11]. The symptoms of gas gangrene are often severe and require a prompt decision for splenectomy. However, the case of a 74-year-old woman on immunosuppressants reported by Hellinckx et al. has improved with percutaneous drainage and antimicrobial therapy [12]. Therefore, drainage may be a treatment option if the patient's condition permits it. As our case had no fluid retention in the spleen and percutaneous drainage was deemed ineffective, a prompt splenectomy was performed, and the patients had a good postoperative course.

## Conclusions

We encountered a case of splenic gas gangrene caused by *C. perfringens* after blunt trauma. The symptoms were severe, but prompt antimicrobial administration and splenectomy improved the patients. *C. perfringens* is a part of the normal gut microbiota, can translocate from the intestine to other parts of the body, and can only grow in an anaerobic environment. Although splenic abscess is caused by many types of pathogens, if an anaerobic environment such as gas production is suspected, a treatment strategy including splenectomy should be considered as soon as possible considering gas gangrene caused by *C. perfringens*.
